# Effect of symbiotic supplementation on glycemic control, lipid profiles and microalbuminuria in patients with non-obese type 2 diabetes: a randomized, double-blind, clinical trial

**DOI:** 10.1186/s40200-017-0304-8

**Published:** 2017-06-02

**Authors:** Zarin sadat Ebrahimi, Ensieh Nasli-Esfahani, Azadeh Nadjarzade, Hassan Mozaffari-khosravi

**Affiliations:** 1 Department of Nutrition, Faculty of Health, Shahid Sadoughi University of Medical Sciences, Bahonar Square, Central Building, Yazd, Iran; 2 Endocrinology and Metabolism Researcher Center, Endocrinology and Metabolism Clinical Sciences Institute, Tehran University of Medical Sciences, Tehran, Iran; 3 Nutrition and Food Security Research Centre, School of Public Health, Shahid Sadoughi University of Medical Sciences, Yazd, Iran; 4 Yazd Diabetic Research Center, Shahid Sadoughi University of Medical Sciences, Yazd, Iran

**Keywords:** Type 2 diabetes, Symbiotic, Probiotic, Microalbuminuria

## Abstract

**Background:**

The prevalent raise of type 2 diabetes (T2D) around the globe, are creating higher risk for cardiovascular diseases (CVDs) and increasing strain on each country’s health care budget in the world. Microalbuminuria has appeared as a key parameter in diabetic patients. Microalbuminuria is also related to increased cardiovascular morbidity in people who are non-obese diabetic. Some studies have suggested that consumption of symbiotic foods might help improve the metabolic profile, inflammatory factors and biomarkers of oxidative stress. The aim of trial was to determine the effect of symbiotic supplementation on glycemic control, lipid profiles and microalbuminuria in non-obese T2D.

**Methods:**

In this randomized, double-blind, clinically controlled trial, 70 patients with T2D (28 females, 42 males) were randomly divided into two groups (*n* = 35 for each group). The symbiotic group (SG) consumed 500 mg/d of symbiotic supplementations containing probiotics (Lactobacillus family, Bifidobacterium family, Streptococus thermophilus), Prebiotics (Fructo oligosaccharide) and B group vitamins (1 mg), lactose (0.5 mg), malt-dextrin, magnesium saturate and the placebo group (PG) consumed capsules filled with row starch and also B group vitamins (1 mg), lactose (0.5 mg), malt-dextrin, magnesium saturate for 9 weeks. Fasting blood glucose (FBG), hemoglobin A1c (HbA1c), blood lipid profiles, 24-h dietary recalls, and anthropometric measurements were measured at the baseline and at the end of trial. SPSS software, version 16 was used to test the data and the results were expressed as mean ± standard deviation. Paired samples T-Test were used to compare continuous variables within groups. Comparison between different groups was performed through two independent samples T-Test. In the absence of normal distribution, the comparison between the groups was made using non-parametric Wilcoxon on signed ranks and Mann–Whitney tests. P values <0.05 was considered significant.

**Results:**

Symbiotic supplementation decreased significantly, FBG (*P* = 0.05) and HbA1c (P < 0.01). There were no significant differences in lipid profiles within and between the groups at the end of study (P > 0.05). Microalbuminuria (P < 0.05) and HbA1c (P < 0.05) are increased significantly in PG at the end of the study. Furthermore, the mean changes of microalbuminuria and HbA1c experienced significant between the two groups. There was significant reduction in urea between two groups from baseline (*P* = 0.051). No significant changes in baseline were shown in creatinine among the two groups or within either groups (P > 0.05).

**Conclusion:**

The consumption of 500 mg/d symbiotic supplementation for 9 weeks could improve the HbA1c, BMI and Microalbuminuria in T2D. Although, No effect has been indicated on FBS, lipid profiles, urea and creatinine.

**Trial Registration:**

The trial has been registered in the Iranian Registry of Clinical Trials IRCT2015072223284N1, identifier. Registered 21 May 2016 “retrospectively registered”.

## Background

Type 2 diabetes (T2D) has been raised quickly in the world during the recent years [1]. Approximately 85– 95% of patients with diabetic patients have T2D. It has been evaluated that 8% of adult in Iran are impressionable [1].

**Fig. 1:**
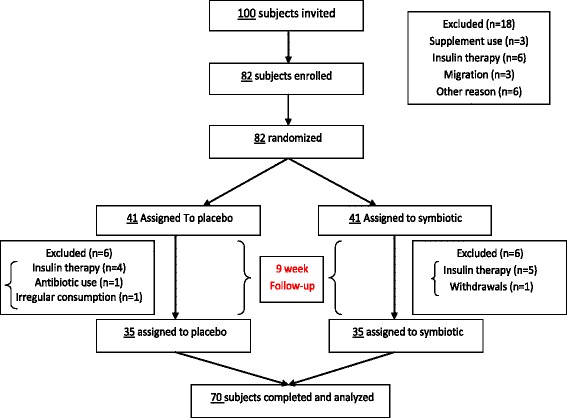
Trial profile

The prevalent raise of T2D around the world are developing higher risk for CVDs and raising species on health care budget of each country in the globe [2]. A specified category of risk factors associated to CVDs and T2D is frequently assigned to as the metabolic or the cardio metabolic disease. The diagnostic risk factors are basically consist hypertriglyceridemia, hyperglycemia, decreased high- to low-density-lipoprotein cholesterol ratio, insulin resistance and in certain descriptions microalbuminuria [3].

Secretion of urinary albumin above20 μg/min (microalbuminuria) is forcefully prognostic of disease and death in patients who suffer from diabetes mellitus, especially T2D. Microalbuminuria has appeared as a key parameter in patients with T2 diabetes [4]. Also, the affiliation between albumin excretion and the risk of mortality is present, when other risk factors as sex, age, weight gain, smoking, ischemic heart disease and increased plasma lipid concentrations are derived from patients [5, 6]. Also, it is a predictor of future infarcts, mortality and aftertime heart failure [7]. Because of the contrary effect of microalbuminuria on improvement in diabetic patients, and the renal risk of macroalbuminuria, testing and intervention plans must be performed primarily, at the microalbuminuria period [8].

Diabetic nephropathy is the usual reason for end stage renal disease happening [9]. In researches on patients with both Insulin Dependent Diabetes Mellitus (IDDM) and Non-Insulin Dependent Diabetes Mellitus (NIDDM), urinary secretion of albumin has been discovered to be predictor of improvement in proteinuria and end-stage DN [10].

In the last years, there has been raising portion in the microorganisms that inhibit the human micro biome [11]. Maximum of these micro biomes inhabit within our gastrointestinal tract, and their number and position are determined by host’s phylogeny and diet [12]. It has been indicated that the gut microbiota combination is related to situations such as allergies, diabetes, cardiovascular disease, intestinal inflammatory disease, cancer and dyslipidaemia [13]. Variations in intestinal microbiota composed with specific nutrition can result in raised intestinal permeability leading via ultimate persistent condition of low-grade inflammation to the advancement of insulin resistance [14]. The function of probiotics in disease states has been studied largely, and the connections between various bacterial groups with insulin resistance, and diabetes are coming to clear [15]. Probiotics are beneficial because they have altered the mixture of the gut bacterial population and characterize the intake of nutrition and appetite [16], and also, they have illustrated useful effects in other diabetes-related situations containing body weight and inflammatory markers [17].

Alterations in the microflora may suppressed production of useful gut hormones such as glucagon-like peptide-1 (GLP-1), increased triglyceride production, insulin sensitivity, inflammation and changes in energy balance. Symbiotics are useful because they have changed the gut flora and increased GLP-1 and GLP-2 hormones. GLP-1 is promoting satiety and lowering glucose levels. Also, GLP-2 (glucagon like-peptide-2) is a proglucagon-derived peptide that decreases intestinal permeability. Secretion of GLP-1 and GLP-2 hormones may be associated with weight reduction, hypoglycemia and reduced HbA1c and lipid profiles [15].

Evidence of last research demonstrated a significant impact of gut microbiota on body’s weight in patients with T2D. The consumption of probiotics has been shown to decrease inflammation and oxidative stress signs, and to recover glycemic and insulin metabolism in non-obese T2 diabetic patients [18].

Experimental data show that intestinal microbiota change with probiotics and prebiotics may positively affect the host’s adiposity and glucose metabolism, but their effects are temporary and they would reduce gently after interruption. Today, it is usual to discover probiotic foods with added prebiotics, like a mixture, in which the concentration of prebiotic is typically below 10 g/kg is known as a ‘symbiotic’. In symbiotics existed the potential synergy between probiotics and prebiotics. Some studies have demonstrated that the usage of symbiotic foods might assist the management of the metabolic indexes, inflammatory agents and biomarkers of oxidative stress [19].

Lastly, A few publications have also explained that consumption of symbiotics may terminate modified insulin sensitivity and metabolic indexes in non-obese T2 diabetic patients [20]. The good affects of symbiotics on insulin metabolism and inflammatory markers could be interceded by the manufacture of short chain fatty acids, modulation of the intestinal microbiota composition, and reduced expression of inflammation-linked genes. Tajadadi et al. showed that the consumption of symbiotic food had advantageous effects on insulin metabolism among patients with diabetes [21].

The mixture of the probiotic and prebiotic foods might improve the living of the bacteria passing the superior section of the gastrointestinal tract, thereby elevating their beneficial effects in the colon [22]. The present study’s purposes are to test the hypothesis that symbiotics can effect on glycemic control, lipid profiles, HbA1c and microalbuminuria in non-obese patients with T2D.

## Methods

### Study design and participants

This study was a single center, randomized, double-blind, placebo-controlled trial. Eighty two patients with T2D (44 men and 38 women) were recruited from diabetic patients of Diabetes & Metabolic Diseases Clinic 1 of Tehran University of Medical Sciences in Tehran, Iran. Recruitment was done by telephone and personally contact. All patients had been diagnosed with T2D for 5 years. T2D was diagnosed according to the criteria of world health organization (World Health Organization, 1985) [23]. These diabetic patients were 35 to 75 years old who were divided to symbiotic (SG) or placebo group (PG) subjects randomly and using a block randomization procedure with matched subjects in each block based on sex and age. The allocation of the SG or PG group was concealed from researchers and the symbiotic and placebo capsules had an identical appearance and packing. Therefore, neither the subjects nor the investigators were aware of the treatment assignments in this double-blinded study. Diabetic patients who have BMI under 35, have microalbuminuria and are consent to participate in the study, are not pregnant or in lactation were included in this study. Patients, who use antibiotic drugs or any new supplementation or drugs, the presence of kidney, liver, or inflammatory intestinal disease, thyroid disorders, immunodeficiency disease, required insulin injections, don’t use symbiotics or placebos more than 10 consecutive days, migrate or die were excluded from this study and also, don’t must be on angiotensin-converting enzyme inhibitor or angiotensin receptor blocker treatment. Each group consisted of 35 patients and they were followed up by telephone once a week.

The sample size was determined based on the primary information obtained from the study by Ejtehad et al. [24].The sample size was computed for α value equal to 0.05 and a power of 80%. Patients in the SG received one symbiotic tablet daily that is receptacle 500 mg probiotic and prebiotic and also, PG received one placebo tablet daily for 9 week. Patients were instructed to keep the symbiotic capsules in the refrigerator and were advised to keep their usual dietary habit, lifestyle, and physical activities and also, to avoid consuming any new drugs and supplementation without researcher’s notice. Arrangement was made so that the patients would receive 15 days supply of their symbiotic capsules every 15 days. The symbiotic capsules contained Probiotics *(Lactobacillus family, Bifidobacterium family, Streptococus thermophilus*), Prebiotics *(*Fructo oligosaccharide) and B group vitamins (1 mg), lactose (0.5 mg), malt-dextrin, magnesium saturate and talc which were produced by Zist takhmir Company. The placebo capsules were filled from row starch and B group vitamins (1 mg), lactose (0.5 mg), malt-dextrin, magnesium saturate and talc. The placebo and symbiotic capsules are identical on similar flavor, odor and appearance.

### Measurements

Information on food consumption, anthropometric measurements, and biochemical analyses (Fasting Blood glucose (FBG), Creatinine, Urea, Triglyceride (TG), Total Cholesterol (TC), HDL, LDL, HbA1c and urine sampling for determining Alb/Cr) were collected at the baseline and after intervention at the end of study. Nutrient intakes during 3 day were estimated using a 24-h food recall at the baseline and at the end of the trial. Three-day average were analyzed by Nutritionist IV software (N squared computing, San Bruno, Calif., USA). Anthropometric measurements were recorded by trained personnel. Body weight was measured using a scale (Seca, Hamburg, Germany) with 0.1 kg accuracy without shoes and with minimum clothing. Height was measured using a stadiometer (Seca) with 0.1 cm accuracy without shoes. BMI was calculated by dividing body weight (kilograms) by height (meters) squared. In the beginning and the end of study, 5 ml blood and 5 ml morning urine sample was drawn for each patient from the antecubital vein in the arm after 12 hour overnight fast.

FBG was measured using the standard enzymatic method with a Parsazmun kit (Karaj, Iran). Urea was measured using the photometric technique and parsazmun kit. Creatinine was measured by colorimetric Jaffe, kinetic. Glycated hemoglobin (HbA1c) was measured in the whole blood by HPLC method. TC and TG were measured by parsazmun kit and photometric method. Low density lipoprotein cholesterol (LDLc) and high density lipoprotein cholesterol (HDLc) were measured photometrical using parsazmun kit. For measuring random microalbuminuria using parsazmun kit and imunoturbidometric method. The total of biochemical analyses were done by equipped laboratory of Diabetes & Metabolic Diseases Clinic 1 of Tehran University of Medical Sciences in Tehran.

### Data analysis

SPSS software, version 16 was used to test the data and the results were expressed as mean ± standard deviation. Paired samples T-Test were used to compare continuous variables within groups. Comparison between different groups was performed through two independent samples T-Test. In the absence of normal distribution, the comparison between the groups was made using non-parametric Wilcoxon on signed ranks and Mann–Whitney tests. *P* values <0.05 was considered significant.

### Ethic consideration

The present study was conducted according to the guidelines laid down in the Declaration of Helsinki and all procedures involving human persons were approved by the ethics committee at Yazd University of Medical Sciences and Health Services. Written informed consent was obtained from all patients. The trial has been registered in the Iranian Registry of Clinical Trials (http://www.irct.ir, identifier: IRCT2015072223284N1 .

## Results

According the Fig. 1; Six patients were excluded from the PG and also, 6 patients were excluded from the study in the SG. Eventually, this study was conducted on 70 patients, of which 28 were females and 42 males (*n* = 35 for each group).According to Table 1, the patients’ baseline characteristics did not differ significantly between the two groups (*P* > 0.05). According to our analysis, there was statistically significant increase in weight at the end of the trial in the PG (*P* = 0.005). BMI had significant differences at the end of study in PG and SG statistically (*P* = 0.012 and *P* = 0.054, respectively). The patients demonstrated good compliance with the capsule consumption and no adverse effects or symptoms were reported (Table 2).

**Table 1:** Characteristics of study participants in baseline and after intervention

Variables	Symbiotic group (*n* = 35)	Placebo group (*n* = 35)	value^a^ρ
Age (years)	58.71 ± 8.20	58.63 ± 8.06	0.96
Sex (male/female)	(23/12)	(19/16)	0.09
Height (cm)	166.16 ± 7.47	165.44 ± 9.71	0.73
Weight (kg) Baseline	77.59 ± 10.54	74.61 ± 11.15	0.25
After	77.50 ± 10.54	74.77 ± 11.16	0.29
BMI (kg/m^2^) Baseline	28.13 ± 3.78	27.30 ± 3.81	0.36
After	28.10 ± 3.78	27.38 ± 3.81	0.43
Metformin (number/day)	2.37 ± 0.59	2.40 ± 0.55	0.83
Glibenclamide (number/day)	1.03 ± 0.74	1.06 ± 0.80	0.87
Glitazine (number/day)	0.57 ± 0.77	0.60 ± 0.73	0.87
Atrovastatine (number/day)	0.89 ± 0.71	0.94 ± 0.68	0.73

Data are presented as mean ± Standard Deviation

BMI = Body Mass Index

^a^Independent sample T-Test

**Table 2:** Compare within two groups (Symbiotic & Placebo groups)

Variables	Symbiotic group (*n* = 35)	Placebo group(*n* = 35)
Weight (kg) value^a^ρ	0.07	0.001>
BMI (kg/m^2^) value^a^ρ	0.05	0.01

BMI = Body Mass Index

^a^Paired T-Test

Table 3 shows dietary intakes of patients throughout the study. As can see, there weren’t any side effects reported following the consumption of symbiotic in patients within this study. The intake of monounsaturated fatty acid was significantly different between the SG and PG at baseline (*P* = 0.023) and the end of the study (*P* = 0.023). No significant difference in energy and other nutrient intakes was observed between the two groups at the beginning and the end of the study (*P* > 0.05).

**Table 3:** daily dietary intakes of patients throughout the study

Variables	Before	After	value^a^ρ
Energy (Kcal/day)			
Placebo group	1656.25 ± 313.98	1677.00 ± 330.14	0.40
Symbiotic group	1746.31 ± 430.06	1742.34 ± 372.62	0.84
ρ value^b^	0.32	0.44	
CHO (g/day)			
Placebo group	264.12 ± 56.53	268.32 ± 55.81	0.38
Symbiotic group	277.94 ± 79.45	272.75 ± 66.11	0.37
ρ value^b^	0.44	0.76	
Pro (g/day)			
Placebo group	84.25 ± 117.69	67.40 ± 17.69	0.39
Symbioti group	68.43 ± 17.53	72.00 ± 19.87	0.12
ρ value^b^	0.43	0.31	
Fat (g/day)			
Placebo group	41.55 ± 15.82	40.57 ± 15.26	0.71
Symbiotic group	46.54 ± 20.46	46.26 ± 18.49	0.87
ρ value^b^	0.25	0.16	
Cholesterol (g/day)			
Placebo group	157.45 ± 153.76	142.21 ± 103.73	0.57
Symbiotic group	175.32 ± 134.00	168.67 ± 117.51	0.65
ρ value^b^	0.60	0.32	
SFA (g/day)			
Placebo group	12.65 ± 3.83	13.34 ± 3.61	0.22
Symbiotic group	14.29 ± 4.71	15.01 ± 4.58	0.28
ρ value^b^	0.11	0.09	
MUFA(g/day)			
Placebo group	11.27 ± 4.26	11.78 ± 4.35	0.39
Symbiotic group	14.44 ± 6.81	14.70 ± 6.00	0.64
ρ value^b^	0.02	0.02	
PUFA (g/day)			
Placebo	6.15 ± 3.88	6.72 ± 4.35	0.21
Symbiotic Supplement	9.45 ± 10.89	8.99 ± 9.83	0.18
ρ value ^b^	0.09	0.21	
Fiber (g/day)			
Placebo group	12.77 ± 6.00	14.97 ± 14.17	0.38
Symbiotic group	12.07 ± 6.31	11.83 ± 5.31	0.73
ρ value^b^	0.63	0.22	
Vitamin E (g/day)			
Placebo group	6.92 ± 13.17	3.00 ± 2.72	0.09
Symbiotic group	4.38 ± 4.72	4.04 ± 4.33	0.32
ρ value^b^	0.28	0.23	
Vitamin C (g/day)			
Placebo group	56.13 ± 30.82	60.46 ± 33.27	0.24
Symbiotic group	51.58 ± 34.91	56.83 ± 40.29	0.32
ρ value^b^	0.56	0.68	
Selenium (g/day)			
Placebo group	0.02 ± 0.02	0.02 ± 0.01	0.76
Symbiotic group	0.02 ± 0.01	0.03 ± 0.01	0.10
ρ value^b^	0.82	0.30	
Sodium(g/day)			
Placebo group	1982.19 ± 1127.23	2177.36 ± 1492.55	0.35
Symbiotic group	2374.75 ± 1328.88	2166.70 ± 866.06	0.41
ρ value^b^	0.18	0.97	
Potassium (g/day)			
Placebo group	1333.90 ± 411.98	1354.86 ± 379.64	0.70
Symbiotic group	1427.55 ± 443.20	1385.84 ± 306.36	0.47
ρ value^b^	0.36	0.70	

Data are presented as mean ± Standard Deviation

^b^: Student t-test; ^a^ Paired T-Test

CHO: carbohydrate Pro: protein SFA: saturated fatty acid MUFA: mono unsaturated fatty acid PUFA: poly unsaturated fatty acid

Table 4 demonstrates the effect of 9 weeks daily consumption of symbiotic and placebo on FBS, Urea, Creatinine, Lipid profiles, HbA1c and MI (urine Alb/Cr). According to Table 4, there was no statistically significant difference in Urea, Creatinine, Triglyceride, Cholesterol total, HDL-C and LDL-C between or within the groups at the end of the study (*P* > 0.05). There was no statistically significant difference in creatinine, HDL, LDL and HbA1c between the two groups at baseline of the study (*P* > 0.05). There was statistically significant difference in urea between the two groups at the baseline of the study (*P* = 0.051). Triglyceride and LDL-C were decreased in the SG compared with the PG, although the differences were not statistically significant (*P* > 0.05). FBG was significantly decreased in the SG (*P* = 0.050) and also, was decreased in the PG but not statistically significantly (*P* = 0.680). HDL-C was increased in the SG compared with the PG (*P* = 0.586 and *P* = 0.287), but wasn’t statistically significantly.

**Table 4:** Comparison mean of FBS, Urea, Creatinine, Lipid profiles, HbA1c and MI in two groups

Variables	Before	After	value^a^ρ	changes
FBG(mg/dl)				
Placebo group	132.74 ± 32.45	130.23 ± 29.58	0.68	2.51 ± 35.97
Symbiotic group	142.34 ± 40.59	132.11 ± 37.85	0.05	10.22 ± 29.79
ρ value^b^	0.27	0.81		0.33
Urea (mg/dl)				
Placebo group	36.80 ± 14.79	37.94 ± 14.57	0.36	1.14 ± 7.30-
Symbiotic group	31.20 ± 7.67	33.25 ± 7.61	0.10	2.05 ± 7.31-
ρ value^b^	0.05	0.09		0.60
Creatinine (mg/dl)				
Placebo group	1.05 ± 0.22	1.03 ± 0.24	0.22	0.02 ± 0.11
Symbiotic group	1.04 ± 0.26	1.05 ± 0.26	0.82	−0.00 ± 0.09
ρ value^b^	0.91	0.73		0.73
Triglycerides (mg/dl)				
Placebo group	122.00 ± 46.14	126.51 ± 60.86	0.62	4.51 ± 54.51-
Symbiotic group	144.94 ± 78.54	130.37 ± 64.59	0.08	14.57 ± 48.02
ρ value^b^	0.14	0.79		0.12
Cholesterol (mg/dl)				
Placebo group	146.09 ± 28.26	149.09 ± 40.82	0.54	3.0 ± 28.63-
Symbiotic group	145.86 ± 30.50	144.74 ± 31.16	0.80	1.11 ± 26.47
ρ value^b^	0.97	0.61		0.53
HDL-C (mg/dl)				
Placebo group	46.74 ± 9.86	45.62 ± 12.49	0.28	1.11 ± 6.09
Symbiotic group	45.65 ± 12.64	46.20 ± 11.00	0.58	0.54 ± 5.84-
ρ value^b^	0.69	0.84		0.25
LDL-C (mg/dl)				
Placebo group	77.74 ± 18.51	78.80 ± 24.63	0.76	1.05 ± 21.09-
Symbiotic group	77.20 ± 18.74	76.37 ± 17.79	0.78	0.83 ± 17.93
ρ value^b^	0.90	0.63		0.68
HbA1C (%)				
Placebo group	7.50 ± 0.87	7.77 ± 1.13	0.01	−0.26 ± 0.60
Symbiotic group	7.44 ± 0.98	7.13 ± 0.89	0.00	0.30 ± 0.46
ρ value^b^	0.76	0.01		0.001>
Urine Alb/Cr (mg/gr)				
Placebo group	62.77 ± 59.69	81.09 ± 81.58	0.02	−18.31 ± 46.78
Symbiotic group	45.39 ± 38.85	34.94 ± 13.11	0.08	10.44 ± 35.26
ρ value^b^	0.15	0.00		0.001>

Data are presented as mean ± Standard Deviation

^a^Paired t-test

^b^Student t-test

FBG: fasting blood glucose LDL-C: low density lipoprotein cholesterol HDL-C: high density lipoprotein cholesterol Urine Alb/Cr: urine albumin/creatin Microalbuminuria

HbA1c increased significantly from the baseline in the PG (*P* = 0.015) and decreased significantly at the end of study in the SG (*P* = 0.000). As presented in Table 4, microalbuminuria (MI) (Urine Alb/Cr) was different in the baseline and the complete the study in two groups.

Microalbuminuria (MI) in SG was decreased after intervention, but wasn’t statistically significant (*P* = 0.089), while MI was significantly increased in the PG at the end of trial (*P* = 0.027).

## Discussion

In the present study we observed significant effect of 500 mg symbiotic supplementation on body weight, BMI, HbA1c and MI in diabetic patients (35 to 75 Y) for nine weeks. However, there was no effect on food intake except for MUFA. Million et al. in a meta-analysis showed that some Lactobacillus species were significantly affiliated with weight corrections in humans and animals: some Lactobacillus species were related to weight gain while other Lactobacillus species were related to weight loss or an anti-obesity effect [25]. Our study is favorable with Million et al. study [25].

Our results are compatible with the recent study by Sewify et al. in 2016 they evaluated the prevalence of urinary tract infection and antimicrobial susceptibility among diabetic patients with controlled and uncontrolled glycemia in Kuwait. They have provided a lot of evidence that the improvement of glycemia in diabetic patients might assist in decreasing the incidence of urinary tract infection in diabetic patients, chiefly in aged subjects [26]. In our study, we have also shown correlation between glycemic reduction and improvement of MI.

Our findings also have been demonstrated significant decrease in BMI in SG, and weight and BMI increased significantly in PG at the end of the study. In a Review of the studies that were done by Burcelin et al.it has been shown that the consumption of probiotics or symbiotics on diabetic patients might be the effect of glycemia decrease, body weight and BMI [12]. These findings are supported our result. Our findings also, showed consumption of symbiotics supplementation for 9 weeks reduced body weight and BMI in patients with T2D significantly.

Recently, a randomized, double-blind, placebo-controlled study was conducted on 20 volunteers (10 placebo group and 10 symbiotic group), aged 50–60 years for 30 days. Their results of the symbiotic group showed a significant increase (p < 0.05) in HDL cholesterol, non-significant reduction (p > 0.05) in total cholesterol and triglycerides and a significant reduction (*p* < 0.05) in FBG and no significant changes were reported in the placebo group [22]. In our study only FBG was significantly decreased in the SG (*P* = 0.050), but there were non-significantly decrease in triglycerides (*p* = 0.082), TC (*P* = 0.805), LDLc (*P* = 0.786) and non-significantly increase in HDLc (*P* = 0.586).

In a systematic review of the clinical evidence that was done by Ruan et al. It hase been shown that probiotic consumption can control glycemic moderately and better. Also, they were expressed that modification of intestinal microbiota by probiotic supplementation can be a procedure for preventing hyperglycemia in clinical action [27]. This study is correspondent with our results. Hence, more research on effect of symbiotic are needed to do in future studies.

There are publications that show probiotics lower LDL cholesterol, however the data is conflicting [28, 29]. Agerholm-Larsen et al. reported that the consumption of yogurt fermented with different probiotics had uncertain effect on LDLc in obese subjects, they also expressed that hypolipidemic effect is dependent on the species of probiotics [30]. Accordingly, in our results there were no significant changes in LDLc in SG and PG at the end of the trial.

Previous studies regarding the antidiabetic properties of symbiotics has been limited to studies about the effects of symbiotics on FBG, insulin resistance or antioxidant status. Tajadadi-Ebrahimi et al. showed that the consumption of symbiotic bread for 8 weeks had advantageous effects on insulin metabolism among patients with diabetes [21]. In another double-blind, clinical trial that was done by Akram Kooshki et al. about the effect of symbiotics on inflammatory markers on patients with T2DM, has expressed consumption of symbiotic once a day for 8 weeks may decreased serum hs-CRP, IL-6 and TNF-α concentration, which are risk factors for CVD diseases [31].

In this study, we investigated effect of symbiotics not only on FBS, triglyceride, cholesterol, LDL, HDL and HbA1c, but also investigated on MI.

The pilot scale trial about the effect of probiotic dietary supplementation in patients with stage 3 and 4 chronic kidney disease has been done in 2009 by Ranganathan et al. in Canada which was performed on 13 subjects, 40 to 70 years old, with chronic kidney disease. Their results indicated that Mean biochemical group (Complete blood counts, serum biochemical testing, creatinine and urine protein) values for all patients either reduced or remained fixed during probiotic bacterial supplementation, but either remained comparatively fixed or significantly raised during placebo supplementation [32], In our results, only urine protein reduction is compatible with Ranganathan et al.’s results, that proteinuria in our study specified by microalbuminuria. Their mechanisms are perhaps suggestive by dihydropyridine calcium channel blockers which are not as impressive as ACE (Angiotensin-Converting-Enzyme) inhibitors or angiotensin receptor blocker (ARB) in reducing albuminuria [8]. High glucose products abnormally high amounts of free radicals by autoxidation of glucose and protein glycation, and oxidative stress has been reported to be a causal agent of tubule interstitial fibrosis in patients with diabetic nephropathy (DN) [33]. In fact, mechanisms of probiotics or symbiotics are challenging with pathogenic microorganisms for intestinal epithelial receptors, which leave useful antimicrobial combinations that activates pathogenic microbes and alter and block toxins [15].

Consonant to our study, several previous studies in humans and animals have suggested that nutrition of probiotic supplemented milk product (dahi/yoghurt) prevented development of diet induced T2D and metabolic disturbances [34, 35]. These studies also indicated that the consumption of probiotic formulations excluded obesity and diabetes in high fat diet fed to mice, and decreased food intake dramatically [2].

In our searches, there was not any publication about effect of probiotics or symbiotics on MI in patients with T2D found. Therefore, we think this study may to be appendage the beginning of studies about the effect of symbiotics on DN.

The present study, indeed, has reported the first evidence of improved microalbuminuria in diabetic patients. This result is by significant decrease in HbA1c and MI between two groups (*P* = 0.011 and *P* = 0.002, respectively).The exact mechanisms involved in the anti diabetic effects of symbiotics has remained unknown. These effects are perhaps partly related to symbiotic effect on gastrointestinal digestion and absorption.

The limitation of this study included its small sample size, short duration of study. Thus, further studies with bigger sample size are needed to confirm the positive effect of symbiotic on management of diabetes, particularly on MI in diabetic patients.

## Conclusion

The consumption of 500 mg/d symbiotic supplementation for 9 weeks, decreased significantly fasting blood glucose, HbA1c, BMI and microalbuminuria in T2D patients. However, there were no significant changes in lipid profiles, urea and creatinine at the end of the study.

## Abbreviations

Alb/Cr: Albumin/creatinin ratio
BMI: Body mass index
DN: Diabetic Nephropathy
FBG: Fasting Blood Glucose
HbA1c: Hemoglobin A1c
HDLc: High Density Lipoprotein cholesterol
hs -CRP: High-sensitivity C-reactive protein
IDDM: Insulin Dependent Diabetes Mellitus
LDLc: Low Density Lipoprotein cholesterol
MI: Microalbuminuria
NIDDM: Non-Insulin Dependent Diabetes Mellitus
PG: Placebo Group
SG: Symbiotic Group
T2D: Type 2 diabetes
TC: Total Cholesterol
TG: Triglyceride
TNF α: Tumor Necrosis Factor α
